# Characterization of SARS-CoV-2 Variants B.1.617.1 (Kappa), B.1.617.2 (Delta), and B.1.618 by Cell Entry and Immune Evasion

**DOI:** 10.1128/mbio.00099-22

**Published:** 2022-03-10

**Authors:** Wenlin Ren, Xiaohui Ju, Mingli Gong, Jun Lan, Yanying Yu, Quanxin Long, Devin J. Kenney, Aoife K. O’Connell, Yu Zhang, Jin Zhong, Guocai Zhong, Florian Douam, Xinquan Wang, Ailong Huang, Rong Zhang, Qiang Ding

**Affiliations:** a Center for Infectious Disease Research, School of Medicine, Tsinghua University, Beijing, China; b School of Life Sciences, Tsinghua University, Beijing, China; c Key Laboratory of Molecular Biology on Infectious Diseases, Ministry of Education, Chongqing Medical University, Chongqing, China; d Department of Microbiology, Boston University School of Medicine, Boston, Massachusetts, USA; e National Emerging Infectious Diseases Laboratories, Boston University, Boston, Massachusetts, USA; f Unit of Viral Hepatitis, CAS Key Laboratory of Molecular Virology and Immunology, Institut Pasteur of Shanghai, Chinese Academy of Sciences, Shanghai, China; g Shenzhen Bay Laboratory, Shenzhen, China; h School of Chemical Biology and Biotechnology, Peking University Shenzhen Graduate School, Shenzhen, China; i Key Laboratory of Medical Molecular Virology (MOE/NHC/CAMS), School of Basic Medical Sciences, Shanghai Medical College, Biosafety Level 3 Laboratory, Fudan University, Shanghai, China; Virginia Polytechnic Institute and State University

**Keywords:** COVID-19, SARS-CoV-2, Delta variant, Kappa variant, B.1.618, ACE2 decoy receptor

## Abstract

Recently, highly transmissible severe acute respiratory syndrome coronavirus 2 (SARS-CoV-2) variants B.1.617.1 (Kappa), B.1.617.2 (Delta), and B.1.618 with mutations within the spike proteins were identified in India. The spike protein of Kappa contains the four mutations E154K, L452R, E484Q, and P681R, and Delta contains L452R, T478K, and P681R, while B.1.618 spike harbors mutations Δ145–146 and E484K. However, it remains unknown whether these variants have alterations in their entry efficiency, host tropism, and sensitivity to neutralizing antibodies as well as entry inhibitors. In this study, we found that Kappa, Delta, or B.1.618 spike uses human angiotensin-converting enzyme 2 (ACE2) with no or slightly increased efficiency, while it gains a significantly increased binding affinity with mouse, marmoset, and koala ACE2 orthologs, which exhibit limited binding with wild-type (WT) spike. Furthermore, the P681R mutation leads to enhanced spike cleavage, which could facilitate viral entry. In addition, Kappa, Delta, and B.1.618 exhibit a reduced sensitivity to neutralization by convalescent-phase sera due to the mutation E484Q, T478K, Δ145–146, or E484K, but remain sensitive to entry inhibitors such as ACE2-Ig decoy receptor. Collectively, our study revealed that enhanced human and mouse ACE2 receptor engagement, increased spike cleavage, and reduced sensitivity to neutralization antibodies of Kappa, Delta and B.1.618 may contribute to the rapid spread of these variants. Furthermore, our results also highlight that ACE2-Ig could be developed as a broad-spectrum antiviral strategy against SARS-CoV-2 variants.

## INTRODUCTION

Since its emergence in late 2019, severe acute respiratory syndrome coronavirus 2 (SARS-CoV-2), which causes the ongoing COVID-19 pandemic, has evolved into several new viral variants of concern (VOC) and variants of interest (VOI) (1–4). SARS-CoV-2 enters host cells by binding angiotensin-converting enzyme 2 (ACE2) in a species-dependent manner (2, 5, 6). For example, murine, New World monkey, and koala versions of ACE2 do not efficiently bind the SARS-CoV-2 spike protein, hindering viral entry into those species (7). In addition, the spike is the target for vaccine and therapeutic antibodies (8, 9), and mutations in spike may potentially alter SARS-CoV-2 transmission, host tropism, pathogenicity, as well as sensitivity to vaccine-elicited antibodies (10–12). For example, the D614G mutation, identified during the earlier stage of the pandemic, promotes spike binding to ACE2, leading to enhanced virus transmission (13, 14). Subsequently, the N501Y mutation found in the B.1.1.7, B.1.351, and B.1.1.28.1 spike proteins has increased the binding affinity between the receptor-binding domain (RBD) and ACE2, increasing viral fitness and infectivity (1, 15, 16). In addition, spike with the N501Y mutation has gained the ability to utilize mouse ACE2 as the receptor to infect the mouse, expanding its host range (12). In addition, K417N and E484K, found in the B.1.351 variant, contribute to evasion of neutralization by multiple monoclonal antibodies (17–19). Thus, as the COVID-19 pandemic continues, it is critical to closely monitor the emergence of new variants, as well as their impact on viral transmission, pathogenesis, and vaccine and therapeutic efficacies.

Recently, the numbers of COVID-19 cases and deaths in India have risen steeply, and the increased spread was associated with newly identified SARS-CoV-2 variants B.1.617 and B.1.618, which have mutated spike proteins (20, 21). B.1.617.1 (Kappa), which carries E154K in the N-terminal domain (NTD) of spike, L452R and E484Q mutations in the RBD of spike, and P681R in proximity to the furin cleavage site, has been designated a variant of interest (VOI) by the World Health Organization (WHO) (https://www.who.int). B.1.617.2 (Delta), which carries L452R and T478K mutations in the RBD of spike, as well as P681R, has been designated a VOC (https://www.who.int). B.1.618 harbors Δ145–146 (deletion of the 145th and 146th residues) and an E484K mutation in the NTD and RBD, respectively. Indeed, some of the mutations in the Kappa, Delta, and B.1.618 have been found in other variants separately. For example, the L452R mutation has been spotted in the B.1.427 and B.1.429 variants, which have enhanced transmissibility and reduced sensitivity to vaccine-elicited antibodies (22, 23). T478K has been seen in Mexican variant B.1.1.519 (24). Also, the E484Q mutation is similar to the E484K mutation found in the B.1.351 variant, which exhibited reduced neutralization by convalescent-phase sera or monoclonal antibodies (17, 25–27). For the Kappa and Delta variants, this is the first time that L452R and E484Q (Kappa)/T478K (Delta) mutations have been found to coexist and the first time that P681R has been observed; for B.1.618, this is the first time the combination of Δ145-146 in the NTD domain and E484K has been observed.

Here, we characterized the spike proteins of Kappa, Delta, and B.1.618 by their ability to utilize different ACE2 orthologs for cell entry and evaluated their sensitivity to convalescent-phase sera and soluble ACE2-Ig decoy receptor.

## RESULTS

### The spike proteins of Kappa, Delta, and B.1.618 gained increased binding affinity with human ACE2 and other orthologs.

**FIG 1:**
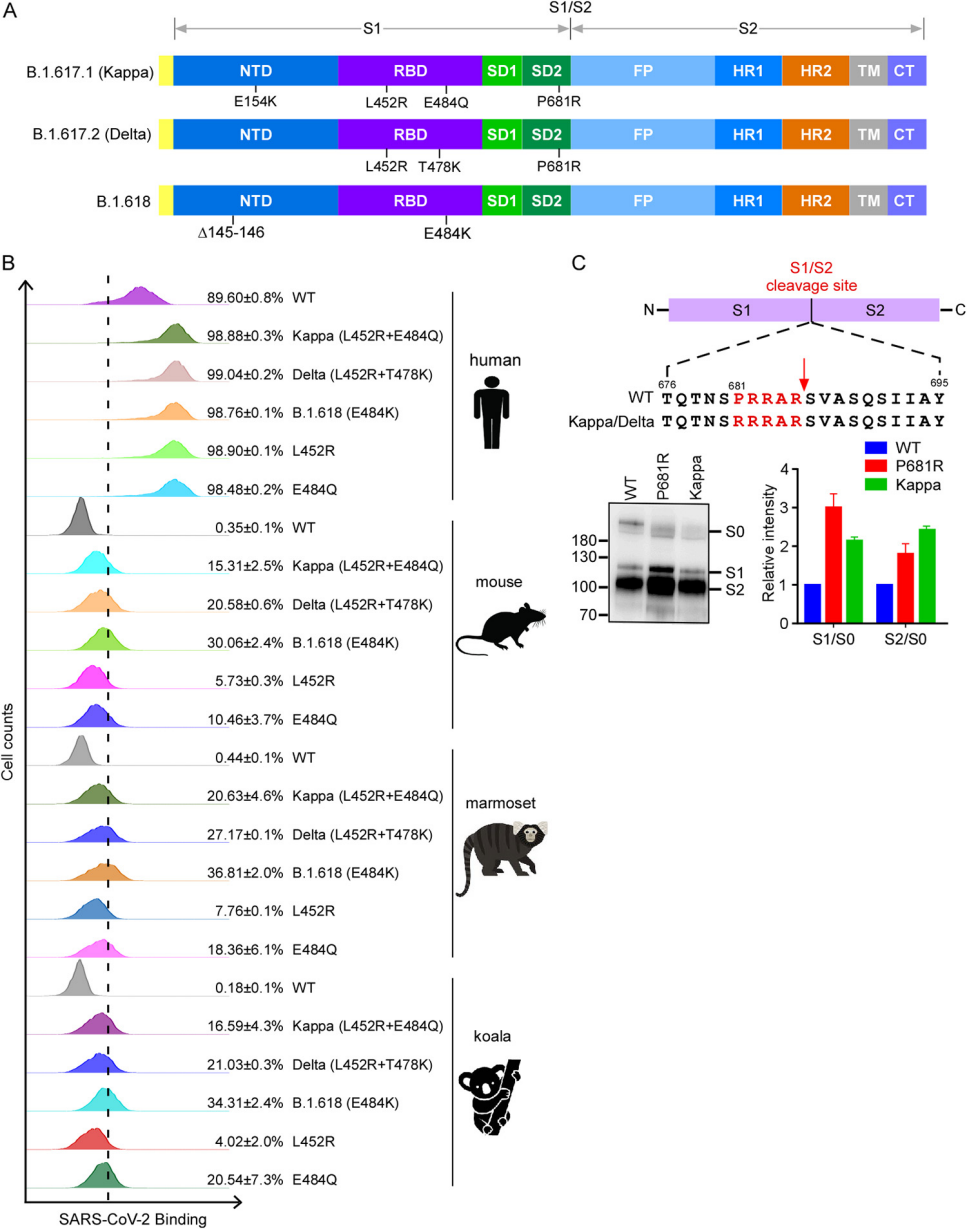
Binding of variants’ spike proteins with ACE2 orthologs and spike protein processing. (A) Schematic overview of SARS-CoV-2 spike proteins of B.1.617.1 (Kappa), B.1.617.2 (Delta), and B.1.618, colored by domain. NTD, N-terminal domain; RBD, receptor binding domain; SD1, subdomain 1; SD2, subdomain 2; FP, fusion peptide; HR1, heptad repeat 1; HR2, heptad repeat 2; TM, transmembrane region; CT, cytoplasmic tail. (B) HeLa-ACE2 cells were incubated with recombinant RBD-His proteins bearing mutations of Kappa, Delta, and B.1.618 or an individual mutation. The binding of RBD-His with cells was analyzed by flow cytometry. Values are expressed as the percentage of cells positive for RBD-His among the ACE2-expressing cells (zsGreen1^+^ cells) and are shown as the means ± standard deviation (SD) from 3 biological replicates. This experiment was independently performed three times with similar results. (C) Immunoblot analysis of spike protein cleavage of pseudovirus of the Kappa and P681R variants using polyclonal antibodies against spike. Full-length spike (S0), S1, and S2 proteins are indicated. The ratio of S1 to S0 or S2 to S0 was quantitatively analyzed using ImageJ software. This experiment was repeated twice independently, and data were normalized to the WT (D614G) of the individual experiment.

The rapid spread of the new emerging variants could be caused by the increased ability to enter the cell, since the variants harbor mutations in the spike proteins (Fig. 1A). To examine the biological impact of these mutations on binding with ACE2 cellular receptor, we employed a cell-based assay that uses flow cytometry to assess the binding of RBD of spike protein to human ACE2 (see Fig. S1A in the supplemental material). We cloned the cDNA of human ACE2 into a bicistronic lentiviral vector (pLVX-IRES-zsGreen1) that expresses the fluorescent protein zsGreen1 via an internal ribosome entry site (IRES) element and can be used to monitor transduction efficiency. Next, wild-type (WT) or variant-derived RBD-His (a purified fusion protein consisting of the RBD and a polyhistidine tag at the C terminus) was incubated with HeLa cells transduced with the human ACE2. Binding of RBD-His to ACE2 was then quantified by flow cytometry (Fig. 1B; Fig. S1). As shown, the binding efficiencies of the RBDs of Kappa (L452R E484Q) (98.88%), Delta (L452R T478K) (99.04%), B.1.618 (E484K) (98.76%), L452R (98.90%), and E484Q (98.48%) were higher than that of the WT (89.6%), suggesting the RBDs of variants bind human ACE2 with a higher affinity (Fig. 1B).

**FIG 2:**
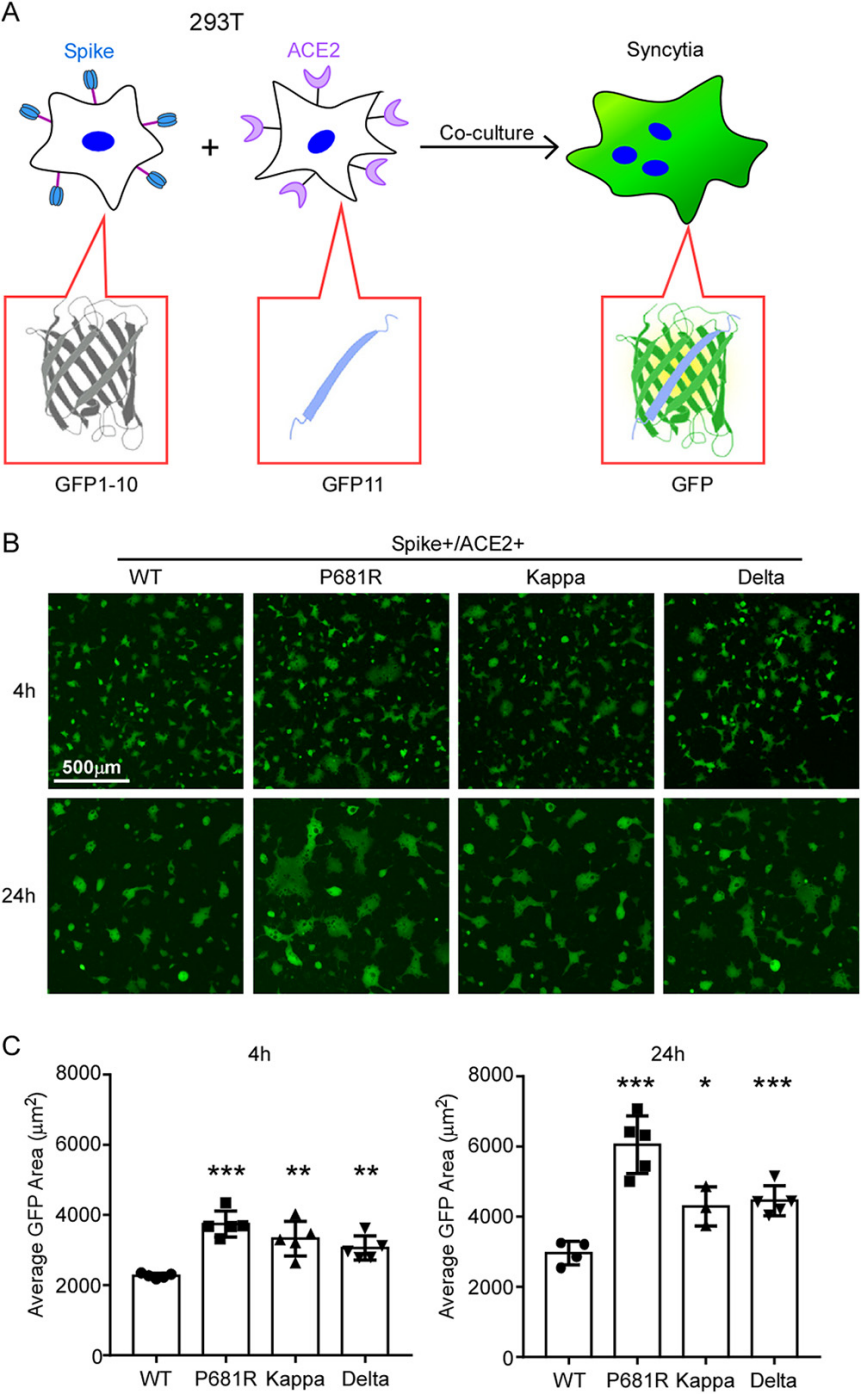
SARS‐CoV‐2 variant spike protein-mediated syncytium formation. (A) Schematic representation of syncytium formation between the donor cells expressing spike protein and split GFP and the acceptor cells expressing ACE2 receptor and split GFP. The images were taken at 4 h and 24 h by Nikon Ti2-U, with least three fields for each condition. The GFP-positive area and fused cell number were measured, and the average GFP areas of syncytia were measured by ImageJ. Results are means ± SD from at least three fields per condition. Results are representative of at least three independent experiments. Scale bar, 500 μm. Statistical analysis was performed by unpaired two-tail *t* test. *, *P* < 0.05; **, *P* < 0.01; ***, *P* < 0.001.

10.1128/mbio.00099-22.1 FIG S1 Gating strategy for determination of the binding efficiency of ACE2 variants with SARS-CoV-2 RBD-His protein. (A) Schematic of testing the efficiency of ACE2 binding with recombinant viral RBD-His protein. (B to E) The main cell population was identified and gated by forward and side scatter. Single cells were further gated by FSC-A and FSC-H. The gated cells were plotted by FITC-A (zsGreen, as the ACE2-expressing population) and APC-A (RBD-His-bound population). The FITC-A-positive cell population was plotted to show the RBD-His-positive population as in Fig. 2B. The binding efficiency was defined as the percentage of RBD-His binding cells among the zsGreen-positive cells. Shown are FACS plots representative of those that have been used for the calculations of binding efficiencies of ACE2 variants with RBD-His. This experiment was independently repeated three times with similar results. Download FIG S1, PDF file, 2.4 MB. Copyright © 2022 Ren et al. 2022 Ren et al. https://creativecommons.org/licenses/by/4.0/ This content is distributed under the terms of the Creative Commons Attribution 4.0 International license.

To test whether the spike proteins of the variants are altered in binding with mouse, marmoset, and koala ACE2 orthologs, we incubated the recombinant RBD-His of the variants’ spike proteins with HeLa cells expressing mouse, marmoset, or koala ACE2, and the binding of RBD-His to the ACE2 ortholog was quantified by flow cytometry (Fig. 1B; Fig. S1). WT RBD-His cannot bind with mouse, marmoset, or koala ACE2, as previously reported (7). In contrast, RBD-His of Kappa, Delta, and B.1.618 binds with mouse, marmoset, and koala ACE2 with various affinities, suggesting that these variants have evolved to gain the function for binding with nonhuman ACE2 orthologs.

Taken together, our results demonstrated that the spike proteins of Kappa, Delta, and B.1.618 have evolved to enhance their binding affinity with human ACE2. Remarkably, the spike proteins of these variants also gain the function to bind with mouse, marmoset, and koala ACE2.

### P681R mutation in Kappa and Delta variants with enhanced spike protein cleavage and increased cell fusion activity.

SARS-CoV-2 spike harbors a multibasic furin cleavage site (residues 681 to 686; PRRARS) at the S1/S2 junction, and the proteolytic processing of the spike by furin and TMPRSS2 proteases is important for SARS-CoV-2 infection (5, 28–31). Kappa and Delta variants contain a P681R substitution (Fig. 1A and C), potentially optimizing the furin cleavage site, which prompted us to examine the effect of the P681R substitution on furin cleavage. To do this, we produced murine leukemia virus (MLV) viral particles pseudotyped with WT, Kappa, or P681R spike. Viruses in the cell culture supernatants were harvested and concentrated for immunoblot analysis of spike protein cleavage by polyclonal antibody against spike protein (Fig. 1C). Interestingly, our data showed significantly increased cleavage of the full-length spike protein (S0) into the S1 and S2 fragments in Kappa spike and P681R spike pseudotyped viruses compared with WT spike. The cleaved S1/S0 ratio was 2.1-fold (Kappa) or 3.0-fold (P681R) higher than that of the WT, and the cleaved S2/S0 ratio was 2.4-fold (Kappa) or 1.8-fold (P681R) higher than that of the WT.

Interaction between spike protein and cellular receptor ACE2 mediated the fusion of virus with host cells, which was a prerequisite of virus entry into host cells (32). To better examine the cell fusion activities of the variants’ spike proteins, we utilized a split green fluorescent protein (GFP) complementation-based cell fusion system to mimic the fusion of SARS-CoV-2-infected cells with ACE2-expressing cells (33, 34). We first derived the HEK293T cells transfected with cDNA expressing split GFP1 to -10 and spike (WT or variants) as the donor cells (HEK293T/GFP1–10/S), and cells transfected with cDNA expressing split GFP11 and human ACE2 as the acceptor cells (HEK293T/GFP11/ACE2). Then, the HEK293T/GFP1–10/S donor cells carrying various spike proteins were cocultured with HEK293T/GFP11/ACE2 acceptor cells at a 1:1 ratio. The donor cells and acceptor cells separately produce half of the reporter protein, generating a fluorescent signal only upon fusion of two cells (Fig. 2A). Microscopy showed that cell fusion occurred rapidly, as evidenced by the appearance of multinucleated GFP^+^ cells (syncytia) at 4 h after the coculture, and the syncytia grew in size as neighbor cells were incorporated in fused cells at 24 h (Fig. 2B). In addition, syncytium formation in the coculture system was dependent on the expression of spike and ACE2 (see Fig. S2 in the supplemental material). The extent of fusion was then quantified by measuring the GFP^+^ area: the spike proteins of the P681R, Kappa, and Delta variants were more active at cell fusion than the WT at both early and late time points (Fig. 2C).

10.1128/mbio.00099-22.2 FIG S2 SARS‐CoV‐2 variants’ spike protein-mediated syncytium formation. Shown is syncytium formation between the donor cells expressing spike protein and split GFP1-10 and the acceptor cells expressing split GFP11 (spike+/ACE2−; left panel) and the donor cells expressing split GFP1-10 and the acceptor cells expressing ACE2 receptor and split GFP11 (spike−/ACE2+; right panel). The images were taken at 4 h and 24 h by Nikon Ti2-U, with least three fields for each condition. The GFP-positive area and fused cell number were measured, and the average GFP areas of syncytia were measured by ImageJ. Results are representative of at least three independent experiments. Scale bar, 500 μm. Download FIG S2, PDF file, 0.1 MB. Copyright © 2022 Ren et al. 2022 Ren et al. https://creativecommons.org/licenses/by/4.0/ This content is distributed under the terms of the Creative Commons Attribution 4.0 International license.

Collectively, these results suggest that P681R substitution in the Kappa and Delta variants could enhance spike cleavage and promote cell fusion.

### Characterization of cell entry driven by spike proteins of Kappa, Delta, and B.1.618.

**FIG 3:**
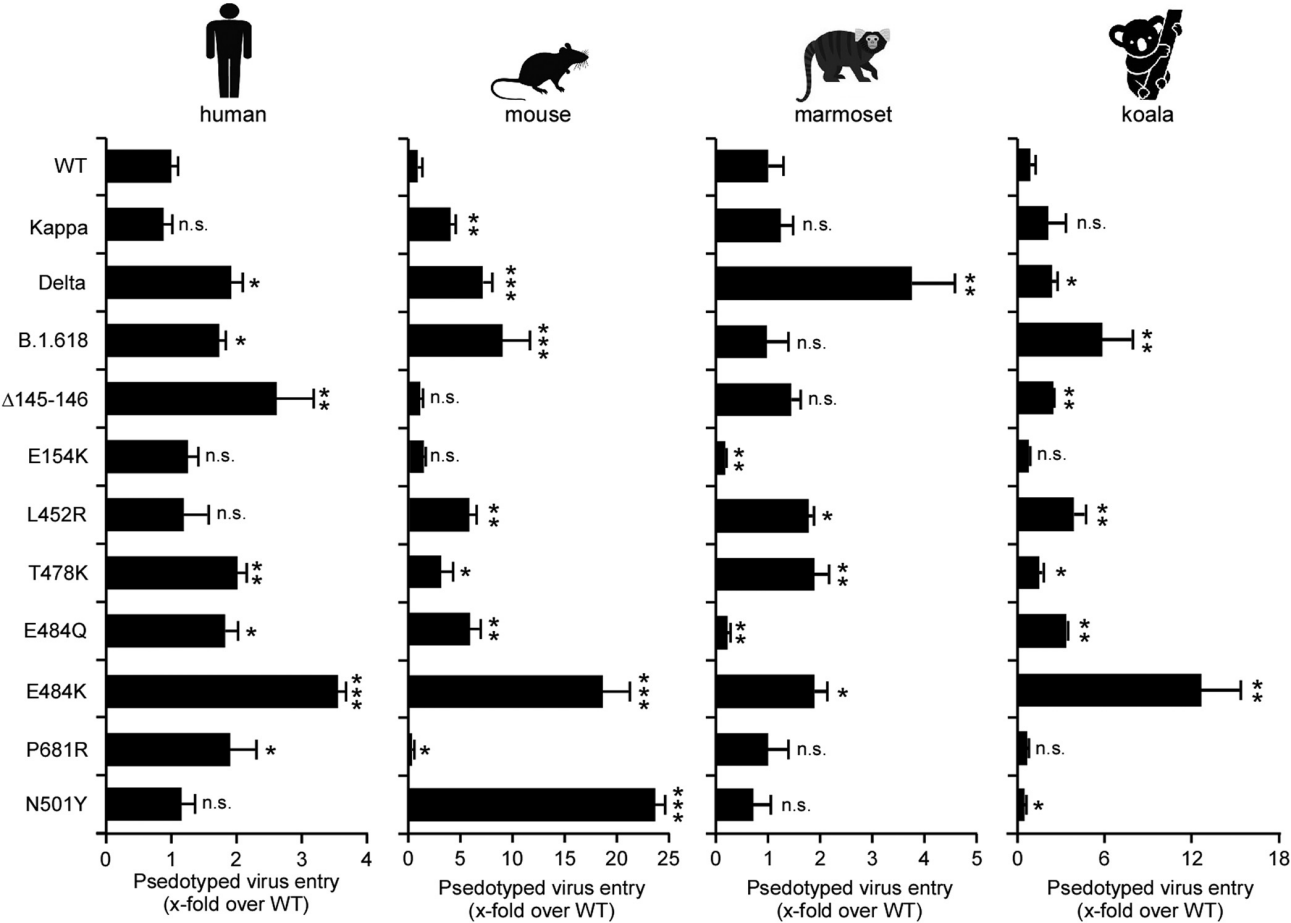
Spike proteins of variant-driven viral entry into HeLa cells expressing human, mouse, marmoset, or koala ACE2 orthologs. Cell entry of the virions pseudotyped with the WT, Kappa, Delta, or B.1.618 spike proteins or individual mutations of these variants’ spike proteins was tested on HeLa cells expressing human, mouse, marmoset, or koala ACE2 orthologs. Luciferase activity was determined after 2 days of infection, and data were normalized to the WT (D614G) of the individual experiment. All infections were performed in triplicate, and the data are representative of three independent experiments (mean ± SD). n.s., no significance; *, *P* < 0.05; **, *P* < 0.01; ***, *P* < 0.001. Significance assessed by one-way ANOVA.

To further examine the biological impact of these mutations on cell entry, we produced pseudotyped virus particles containing a firefly luciferase reporter gene and expressing on their surface with the spike proteins of WT (D614G), Kappa, Delta, and B.1.618 variants. HeLa cells expressing human ACE2 (HeLa-human ACE2) were then inoculated with these pseudoparticles, and at 48 h postinoculation, the cells were lysed and the luciferase activity was monitored as a measure of virus entry (Fig. 3). Compared to WT spike, Delta and B.1.618 spike proteins gained an increased ability to mediate viral entry into HeLa-human ACE2 cells, which was contributed to by the T478K (Delta), P681R (Delta), Δ145-146 (B.1.618), and E484K (B.1.618) variants. The spike protein of Kappa exhibited comparable ability to mediate viral entry: even an E484Q or P681R mutation in its spike could significantly promote viral entry individually.

SARS-CoV-2 has a broad host range, and its spike could utilize a diverse range of ACE2 orthologs for cell entry (7, 35). However, we and others previously found that SARS-CoV-2 spike has a limited binding affinity with mouse, New World monkey, or koala ACE2 and does not efficiently mediate virus entry into these species (3, 7, 36). We thus sought to evaluate the abilities of the variants’ spike proteins to utilize these ACE2 proteins for cell entry. To this end, we produced virus pseudotyped with SARS-CoV-2 variant spike proteins with a single mutation or a combination of mutations. The HeLa cells expressing mouse, marmoset (New World monkey), or koala ACE2 orthologs were then inoculated with pseudoparticles, and luciferase activity was determined at 48 h postinoculation (Fig. 3). Our results showed that spike proteins from Kappa, Delta, and B.1.618 could significantly enhance cell entry into HeLa-mouse ACE2 cells, as a result of the T478K (Delta), E484Q (Kappa), and E484K (B.1.618) mutations. Besides HeLa-mouse ACE2, Delta also exhibited significantly enhanced cell entry into HeLa-marmoset and HeLa-koala cells, which are contributed to by L452R and T478K mutations. In contrast, Kappa did not increase cell entry into HeLa-marmoset or HeLa-koala cells, and B.1.618 only showed enhanced entry into HeLa-koala cells, which is attributable to E484K. In addition, the N501Y mutation in the spike has been demonstrated to have gained the ability to utilize mouse ACE2 for cell entry (12), and our data suggested that the N501Y mutant specifically gained the ability to utilize mouse ACE2 for cell entry, with an efficiency approximately 4-fold higher than that of Delta; however, the N501Y mutation has a negligible effect on utilization of marmoset or koala ACE2 for cell entry.

Taken together, our results demonstrated that the spike proteins of Kappa, Delta, or B.1.618 with distinct mutations have altered their ability to utilize ACE2 orthologs for cell entry. The Delta and B.1.618 variants gained an enhanced ability to use human ACE2 receptor for cell entry. Remarkably, the Delta variant gained the function to utilize mouse, New World monkey, or koala ACE2 orthologs, which cannot be engaged with WT virus, for cell entry.

### Kappa, Delta, and B.1.618 variants exhibited resistance to neutralization by convalescent-phase serum, while remaining sensitive to an ACE2-based decoy receptor antiviral countermeasure.

**FIG 4:**
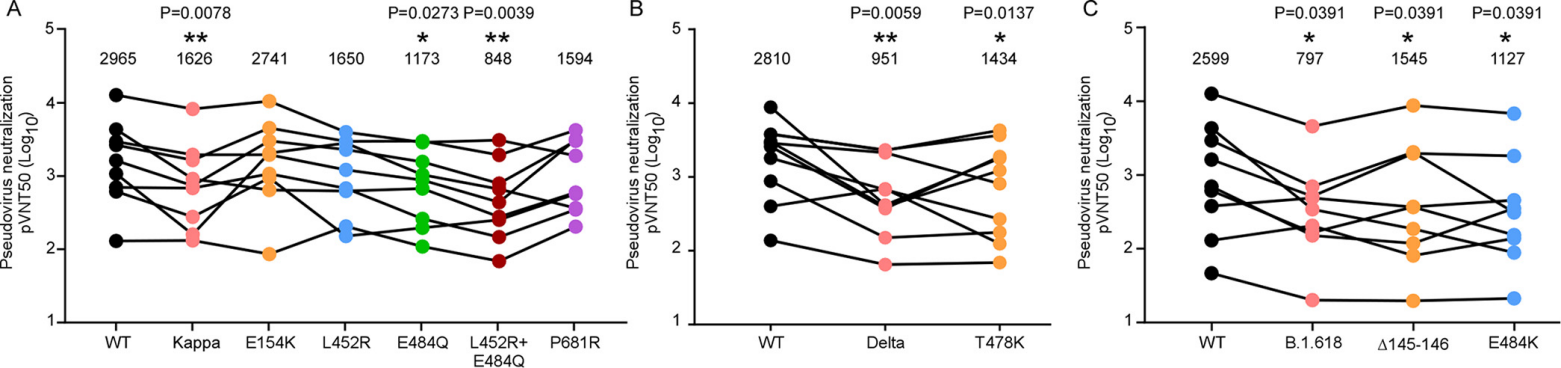
Reduced sensitivity of Kappa, Delta, and B.1.618 to neutralization of convalescent-phase sera. (A to C) MLV particles pseudotyped with the indicated spike proteins of Kappa, Delta, and B.1.618 or mutations as indicated were preincubated with serially diluted convalescent-phase sera, respectively. HeLa-ACE2 cells were incubated with these preincubated mixtures and analyzed 48 h later by measuring luciferase activity to calculate the plasma dilution factor leading to 50% reduction in spike protein-driven cell entry (50% neutralizing titer [NT_50_]). The NT_50_ of each serum against each pseudovirion (pvNT50) is presented, and identical serum samples are connected with lines. Statistical significance of differences between WT and variant spike proteins was analyzed by two-sided Friedman test with Dunn’s multiple comparison. *, *P* < 0.05; **, *P* < 0.01. The pVNT50 of each sample was tested with two repeats.

SARS-CoV-2 infection-elicited neutralizing antibodies target the spike protein, which is critical for protection from reinfection (37, 38). We hypothesized that mutations in the spike protein of the Kappa, Delta, and B.1.618 variants might contribute to the evasion of neutralizing antibodies. Therefore, we sought to determine the sensitivity of these variants to neutralization by convalescent-phase serum. We chose plasma from COVID-19 patients (see Tables S1, S2, and S3 in the supplemental material) and measured the neutralization activity of convalescent plasma against virions pseudotyped with single or combined mutations from the Kappa, Delta, and B.1.618 variants (Fig. 4A to C). To this end, we preincubated the serial-diluted convalescent-phase sera with virion pseudotyped with spike proteins as describe above, and subsequently tested on HeLa-human ACE2 cells. Cell entry of pseudotyped virion in the presence of convalescent plasma with various concentrations was assessed 48 h later by measurement of luciferase activities. The results showed that Kappa, Delta, and B.1.618 exhibited 1.8-, 3.0-, and 3.3-fold resistance to neutralization by convalescent-phase sera, respectively, which is conferred by E484Q, L452R E484Q, T478K, Δ145–146, and E484K (Fig. 4A, B, and C).

10.1128/mbio.00099-22.3 TABLE S1 Summary of serum information of COVID-19 patients for evaluation of Kappa sensitivity. Download Table S1, XLSX file, 0.01 MB. Copyright © 2022 Ren et al. 2022 Ren et al. https://creativecommons.org/licenses/by/4.0/ This content is distributed under the terms of the Creative Commons Attribution 4.0 International license.

10.1128/mbio.00099-22.4 TABLE S2 Summary of serum information of COVID-19 patients for evaluation of Delta sensitivity. Download Table S2, XLSX file, 0.01 MB. Copyright © 2022 Ren et al. 2022 Ren et al. https://creativecommons.org/licenses/by/4.0/ This content is distributed under the terms of the Creative Commons Attribution 4.0 International license.

10.1128/mbio.00099-22.5 TABLE S3 Summary of serum information of COVID-19 patients for evaluation of B.1.618 sensitivity. Download Table S3, XLSX file, 0.01 MB. Copyright © 2022 Ren et al. 2022 Ren et al. https://creativecommons.org/licenses/by/4.0/ This content is distributed under the terms of the Creative Commons Attribution 4.0 International license.

**FIG 5:**
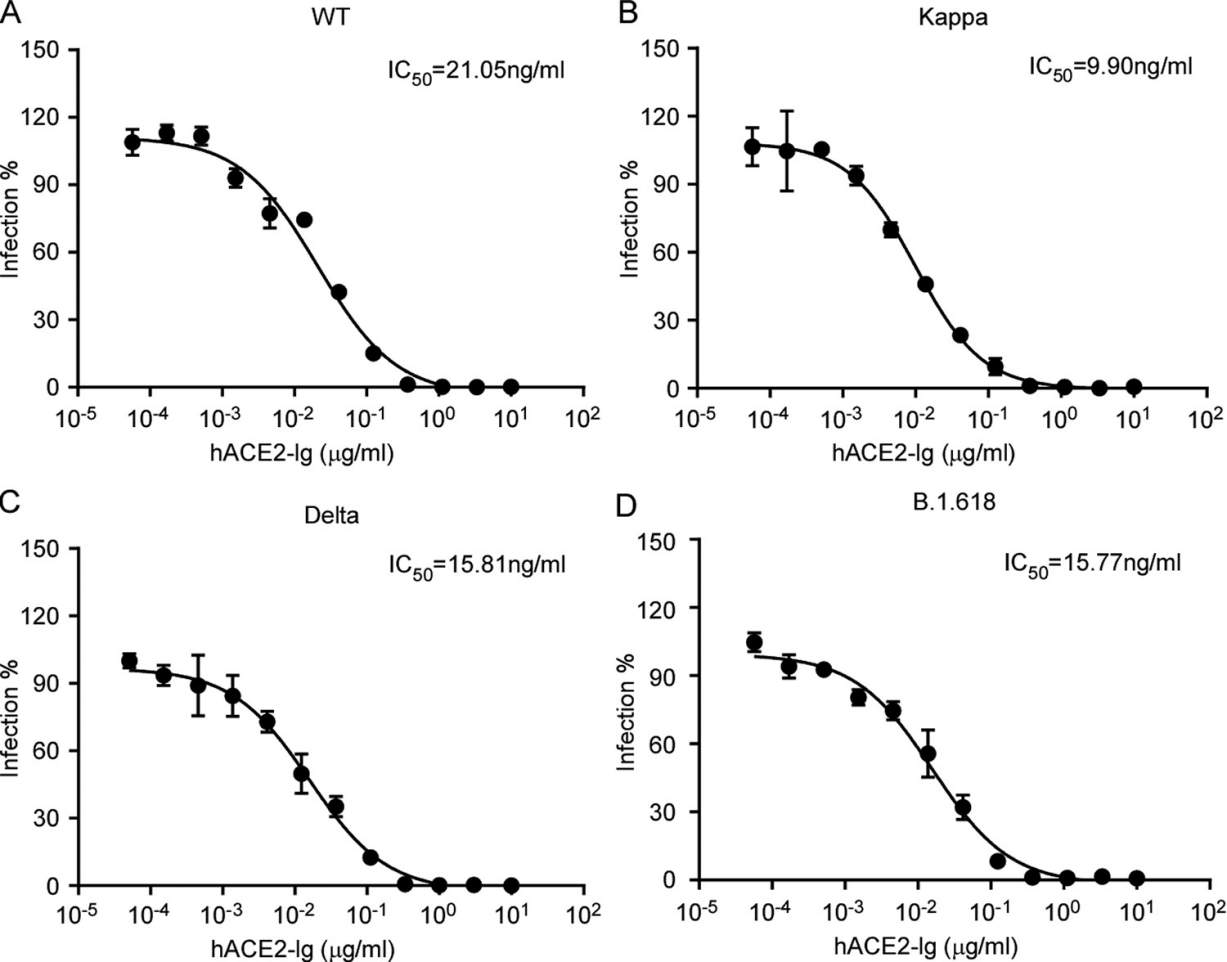
Inhibition of WT, Kappa, Delta, and B.1.618 by recombinant ACE2-Ig decoy receptor. Recombinant ACE2-Ig was diluted at the indicated concentrations. Viral entry was determined by assessing GFP expression 48 h postinfection of the WT (A) or Kappa (B), Delta (C) and B.1.618 (D) trVLP. The dilution factors leading to 50% reduction of trVLP entry were calculated as the IC_50_ by using GraphPad Prism software. The data shown are representative of three independent experiments with similar results, and data points represent the mean ± SD in triplicate.

Previous studies have shown that ACE2-Ig (ACE2 fused with Fc recombinant protein) exhibited a potent antiviral effect against SARS-CoV-2 infection (35, 39). As the spike proteins of Kappa, Delta, and B.1.618 exhibited enhanced ACE2 binding affinity, it could be more sensitive to the inhibition by ACE2 decoy receptor. To this end, we used an SARS-CoV-2 transcription- and replication-competent virus-like particle (trVLP) cell culture system, which recapitulates the entire viral life cycle in Caco-2-N cells, to engineer the desired mutations in the spike proteins of Kappa, Delta, and B.1.618 variants into an SARS-CoV-2 isolate, Wuhan-Hu-1, with a D614G (WT) backbone, and examined the sensitivity of trVLP of Kappa, Delta, and B.1.618 to inhibition of ACE2-Ig. Specifically, we inoculated the Caco-2-N cells with WT, Kappa, Delta, or B.1.618 trVLP (multiplicity of infection [MOI] of 0.1) in the presence of ACE2-Ig at various concentrations. After 48 h, the cells were collected, and GFP expression was quantified as the proxy of virus infection by flow cytometry. ACE2-Ig could potently inhibit WT, Kappa, Delta, and B.1.618 trVLP infection, with 50% inhibitory concentrations (IC_50_s) of 21.05, 9.90, 15.81, and 15.77 ng/mL, indicating that the Kappa, Delta, and B.1.618 variants are still sensitive to inhibition by ACE2-Ig (Fig. 5A, B and C). In summary, the Kappa, Delta, and B.1.618 variants exhibited a reduced sensitivity to neutralization by convalescent-phase serum, while remaining sensitive to the ACE2 decoy receptor antiviral countermeasure.

## DISCUSSION

The emergence of SARS-CoV-2 variants imposes challenges on control of the COVID-19 pandemic (1, 40, 41). The recent surge in COVID-19 cases and mortalities in India is associated with new SARS-CoV-2 variants Kappa, Delta, and B.1.618 with mutated spike proteins (21). In this article, we characterized the biological properties of these new variants, including the efficiency of entry into cells, the binding affinities with human ACE2, as well as other orthologs, and the sensitivity to neutralization by convalescent plasma and recombinant ACE2-Ig decoy receptor.

We found that the Kappa variant did not show increased cell entry of HeLa-human ACE2 cells and had only slightly increased cell entry of HeLa-mouse ACE2 cells. The Delta variant had significantly increased cell entry of HeLa-human ACE2, HeLa-mouse ACE2, HeLa-marmoset ACE2, and HeLa-koala ACE2 cells; consistently, the Delta RBD bound human ACE2 with a higher affinity. Remarkably, Delta RBD gained the ability to bind with mouse, marmoset, and koala ACE2 orthologs, which exhibited limited binding affinity with WT RBD. The B.1.618 variant had dramatically increased cell entry of HeLa-human ACE2, HeLa-mouse ACE2, and HeLa-koala ACE2 cells, but not HeLa-marmoset ACE2 cells. Additionally, the P681R mutation in the spike proteins of Kappa and Delta variants enhanced the spike processing and cell fusion activities (Fig. 1C and Fig. 2A to C). Recent studies of infection with the Delta variant bearing P681R showed it could form large syncytia compared to other variants, further indicating that the P681R mutation in the furin cleavage site could enhance viral fusogenicity. As the furin cleavage site is critical for viral pathogenesis and transmission, the Kappa and Delta variants bearing the optimized furin cleavage site have potentially evolved increased pathogenicity and transmissibility, which urgently needs to be investigated. Remarkably, our results suggest that the Kappa, Delta, and B.1.618 variants have extended their usage of ACE2 orthologs into mice, koalas, and New World monkeys (Fig. 3), raising a potential risk of mice or other rodents becoming the reservoirs for SARS-CoV-2, and the virus could potentially spill back to humans as the mice live close to humans.

SARS-CoV-2 variants Kappa, Delta, and B.1.618 exhibited reduced sensitivity to neutralization by polyclonal antibodies in the serum from individuals previously infected with SARS-CoV-2 (Fig. 4A to C). Our analysis suggests that the immune escape is mainly conferred by the E484Q, T484K, Δ145–146, and E484K mutations: these new variants must be further surveyed to avoid fast spread, and their presence might raise alerts if they might be considered likely to soon contribute to accelerating the spread of the virus in human populations. Thus, our findings highlight the critical need for broad-spectrum neutralizing antibodies insensitive to substitutions arising in VOC or VOI. In addition, we further demonstrated that the ACE2 decoy receptor-based antiviral strategy is an alternative countermeasure against the VOC, as the VOC Kappa and B.1.618 exhibited increased binding with human ACE2 (1, 41). Our results show that recombinant ACE2-Ig protein could inhibit Kappa, Delta, and B.1.618 infection with efficacy comparable to or better than that of the WT (Fig. 5A to D), which is consistent with the results that Kappa, Delta, and B.1.618 spike proteins exhibited increased binding affinity with human ACE2 (Fig. 1B). In addition, it has been demonstrated that human ACE2 peptidase activity and viral receptor activity could be uncoupled; thus, it is possible that enzymatically inactivated ACE2 could be developed as an antiviral in clinics to avoid the potential ACE2 side effects mediated by its enzymatic activity.

New variants of concern will continue to emerge as the COVID-19 pandemic persists, which highlights the importance of genomic surveillance for the early identification of future variants. The potential of variants to escape naturally induced and vaccine-elicited immunity makes the development of next-generation vaccines that elicit broadly neutralizing activity against current and future variants a priority (22, 25, 42). In addition, the suppression of viral replication through both public health measures and the equitable distribution of vaccines, increasing the proportion of the population immunized with the current safe and effective authorized vaccines, is critical to minimize the risk of emergence of new variants. Also, the development of broad-spectrum antivirals, especially against diverse SARS-CoV-2 variants, is therefore of continued significance.

## MATERIALS AND METHODS

### Cell culture.

HEK293T (American Tissue Culture Collection, ATCC, Manassas, VA, CRL-3216), Vero E6 (Cell Bank of the Chinese Academy of Sciences, Shanghai, China), and A549 (ATCC) cells were maintained in Dulbecco’s modified Eagle’s medium (DMEM) (Gibco, NY, USA) supplemented with 10% (vol/vol) fetal bovine serum (FBS), 10 mM HEPES, 1 mM sodium pyruvate, 1× nonessential amino acids, and 50 IU/mL penicillin-streptomycin in a humidified 5% (vol/vol) CO_2_ incubator at 37°C. Cells were tested routinely and found to be free of mycoplasma contamination.

### Plasmids.

The cDNAs encoding the ACE2 orthologs were synthesized by GenScript and cloned into the pLVX-IRES-zsGreen1 vector (catalog no. 632187; Clontech Laboratories, Inc.) with a C-terminal FLAG tag. Spike mutants were generated by Quikchange (Stratagene) site-directed mutagenesis. All constructs were verified by Sanger sequencing.

### Lentivirus production.

Vesicular stomatitis virus G protein (VSV-G) pseudotyped lentiviruses expressing ACE2 orthologs tagged with FLAG at the C terminus were produced by transient cotransfection of the third-generation packaging plasmids pMD2G (Addgene catalog no. 12259) and psPAX2 (Addgene catalog no. 12260) and the transfer vector with VigoFect DNA transfection reagent (Vigorous) into HEK293T cells. The medium was changed 12 h posttransfection. Supernatants were collected at 24 and 48 h after transfection, pooled, passed through a 0.45-μm-pore filter, and frozen at −80°C.

### Surface ACE2 binding with RBD-His assay.

HeLa cells were transduced with lentiviruses expressing the ACE2 variants for 48 h. The cells were collected with TrypLE (Thermo catalog no. 12605010) and washed twice with cold phosphate-buffered saline (PBS). Live cells were incubated with the recombinant proteins’ RBD-His with mutations (Sino Biological catalog no. 40592-V08B, 40592-V08H88, 40592-V08H90, 40592-V08H84, 40592-V08H28, and 40592-V08H81) at 1 μg/mL at 4°C for 30 min. After washing, cells were stained with Anti-His–phycoerythrin (PE) (clone GG11-8F3.5.1; Miltenyi Biotec catalog no. 130-120-787) for 30 min at 4°C. Cells were then washed twice and subjected to flow cytometry analysis (Attune NxT; Thermo). Binding efficiencies are expressed as the percentage of cells positive for RBD-His among the zsGreen-positive cells (ACE2-expressing cells).

### Cell-cell fusion assay.

For HEK293T donor cells (HEK293T/GFP1–10/S), the HEK293T cells were grown to 50% confluence in a 24-well plate and transfected with 0.5 μg pcDNA3.1-GFP1–10 and 0.5 μg pcDNA-SARS-CoV-2 spike (WT or variant). For HEK293T acceptor cells (HEK293T/GFP11/ACE2), the HEK293T cells in a 24-well plate were transfected with 0.5 μg pcDNA3.1-GFP11 and 0.5 μg pLVX-human ACE2-IRES-Puro. After 12 h, the donor cell and acceptor cell populations were trypsinized and seeded together into 24-well plates at a 1:1 ratio. The cells were then left to fuse, and fluorescence images were taken at the indicated time point using a Nikon Ti2-U with at least three fields for each condition.

### Production of SARS-CoV-2 pseudotyped virus, determination of viral entry efficiency, and analysis of spike protein cleavage.

Pseudoviruses were produced in HEK293T cells by cotransfecting the retroviral vectors pTG-MLV-Fluc, pTG-MLV-Gag-pol, and pcDNA3.1 expressing SARS-CoV-2 spike gene or VSV-G (pMD2.G; Addgene catalog no. 12259) by using VigoFect (Vigorous Biotechnology). At 48 h posttransfection, the cell culture medium was collected for centrifugation at 3,500 rpm for 10 min, and then the supernatant was subsequently aliquoted and stored at −80°C for further use. Virus entry was assessed by transduction of pseudoviruses in cells expressing an ACE2 ortholog in 48-well plates. After 48 h, intracellular luciferase activity was determined using the Promega luciferase assay system (Promega catalog no. E1500) according to the manufacturer’s instructions. Luminescence was recorded on a GloMax Discover system (Promega). To analyze the spike protein cleavage, the concentrated pseudoviruses were produced by ultracentrifugation at 100,000 × *g* for 2 h over a 20% sucrose cushion. Western blot detection of SARS-CoV-2 spike protein was performed using a polyclonal spike antibody (Sino Biological catalog no. 40589-T62).

### Human convalescent-phase serum and neutralization of pseudotyped virion particles.

We obtained convalescent-phase serum from COVID-19 patients (Tables S1, S2, and S3) more than 1 month after documented SARS-CoV-2 infection in the spring of 2020. Each plasma sample was heat inactivated (56°C, 30 min) and then assayed for neutralization against WT or Kappa, Delta, and B.1.618 variant pseudoviruses. For neutralization experiments, S protein-bearing pseudotyped virion particles were preincubated for 1 h at 37°C with diluted plasma samples obtained from convalescent COVID-19 patients, before the mixtures were inoculated onto HeLa-ACE2 cells. Transduction efficiency was determined at 48 h postinoculation. This study was approved by the Institution Review Board of Tsinghua University (20210040).

### Recombinant ACE2-Ig protein expression and purification.

ACE2-Ig, a recombinant Fc fusion protein of soluble human ACE2 (residues Gln18 to Ser740) was expressed in 293F cells and purified using protein A affinity chromatography as described in our previous study (35).

### Production of SARS-CoV-2 trVLP.

The desired mutations in the spike proteins of Kappa, Delta, and B.1.618 variants in an SARS-CoV-2 isolate, Wuhan-Hu-1, with a D614G (WT) backbone and the trVLP, were generated as previously described (43).

### Statistical analysis.

One-way analysis of variance (ANOVA) with Tukey’s honestly significant difference (HSD) test was used to test for statistical significance of differences between the different group parameters. *P* values of <0.05 were considered statistically significant.
