# Supplementary figures and images for: Characterization of SARS-CoV-2 Variants B.1.617.1 (Kappa), B.1.617.2 (Delta), and B.1.618 by Cell Entry and Immune Evasion
Source: mBio. 2022 Mar 10;13(2):e00099-22. doi: 10.1128/mbio.00099-22 (PMC9040861; doi:10.1128/mbio.00099-22)

Supplemental Fig 1

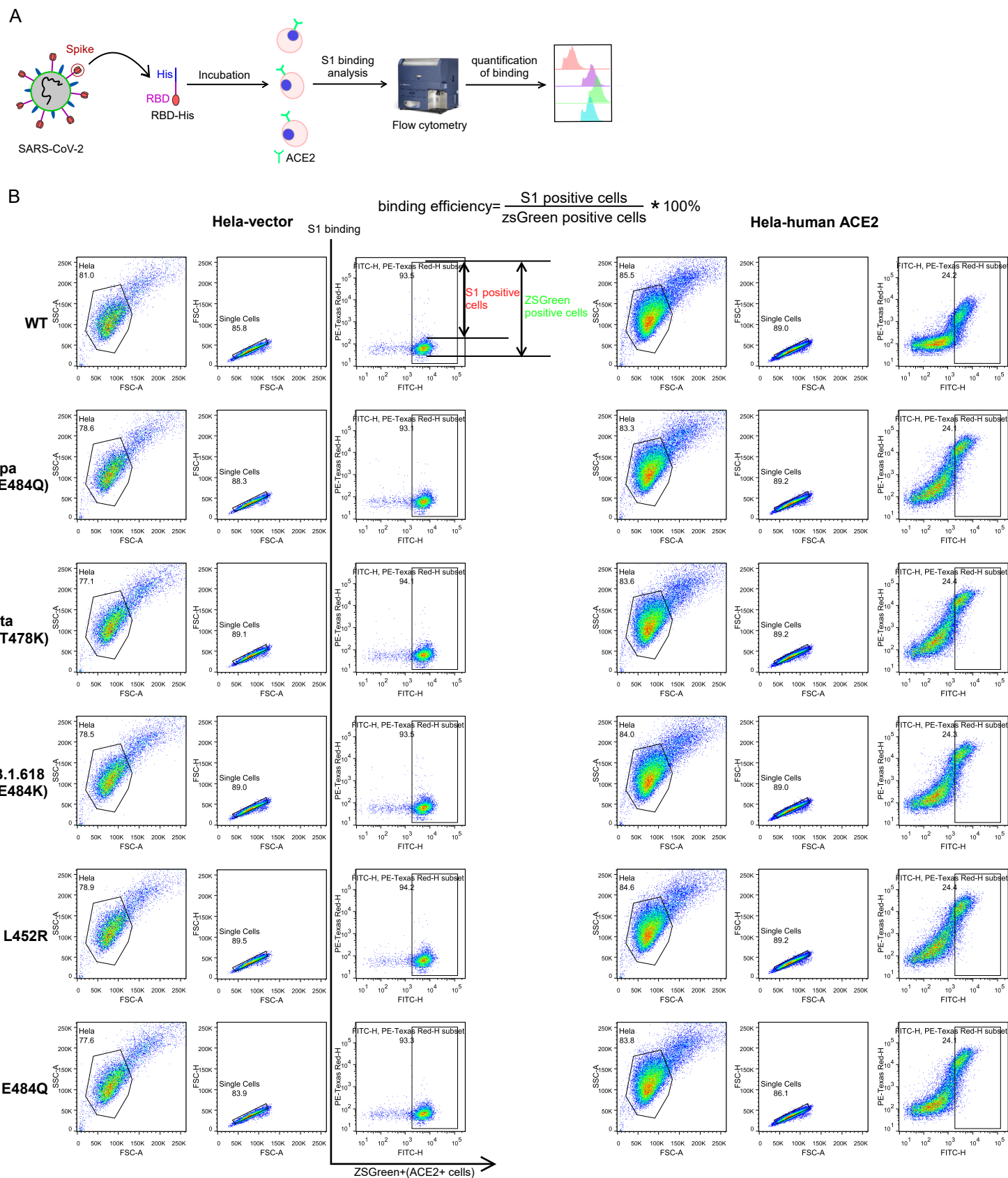

C

## Hela-mouse ACE2

S1 binding

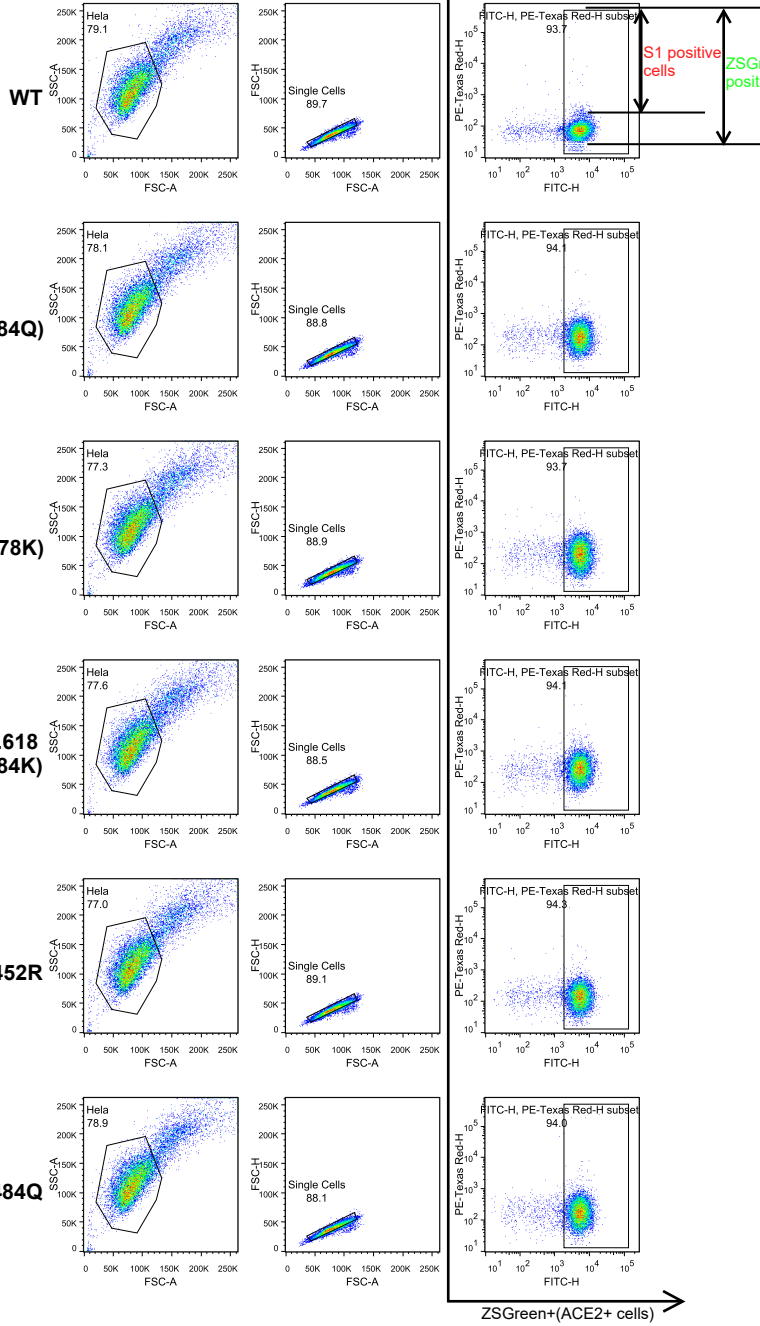

D

## Hela-marmoset ACE2

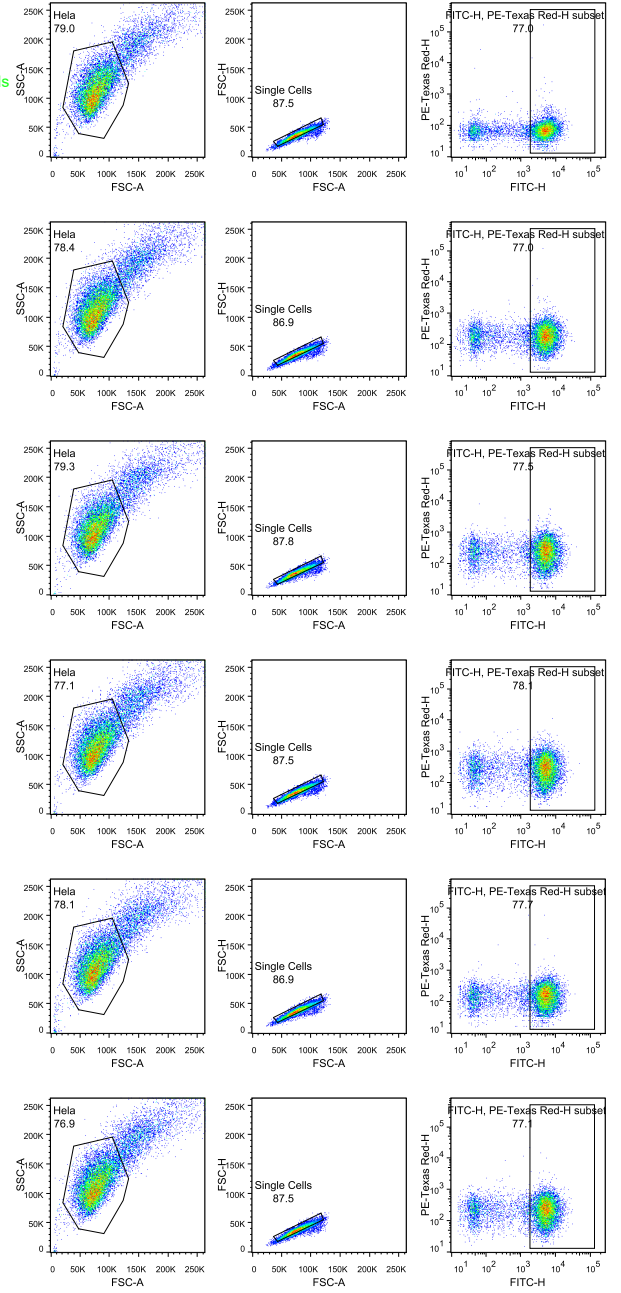

E

# Hela-koala ACE2

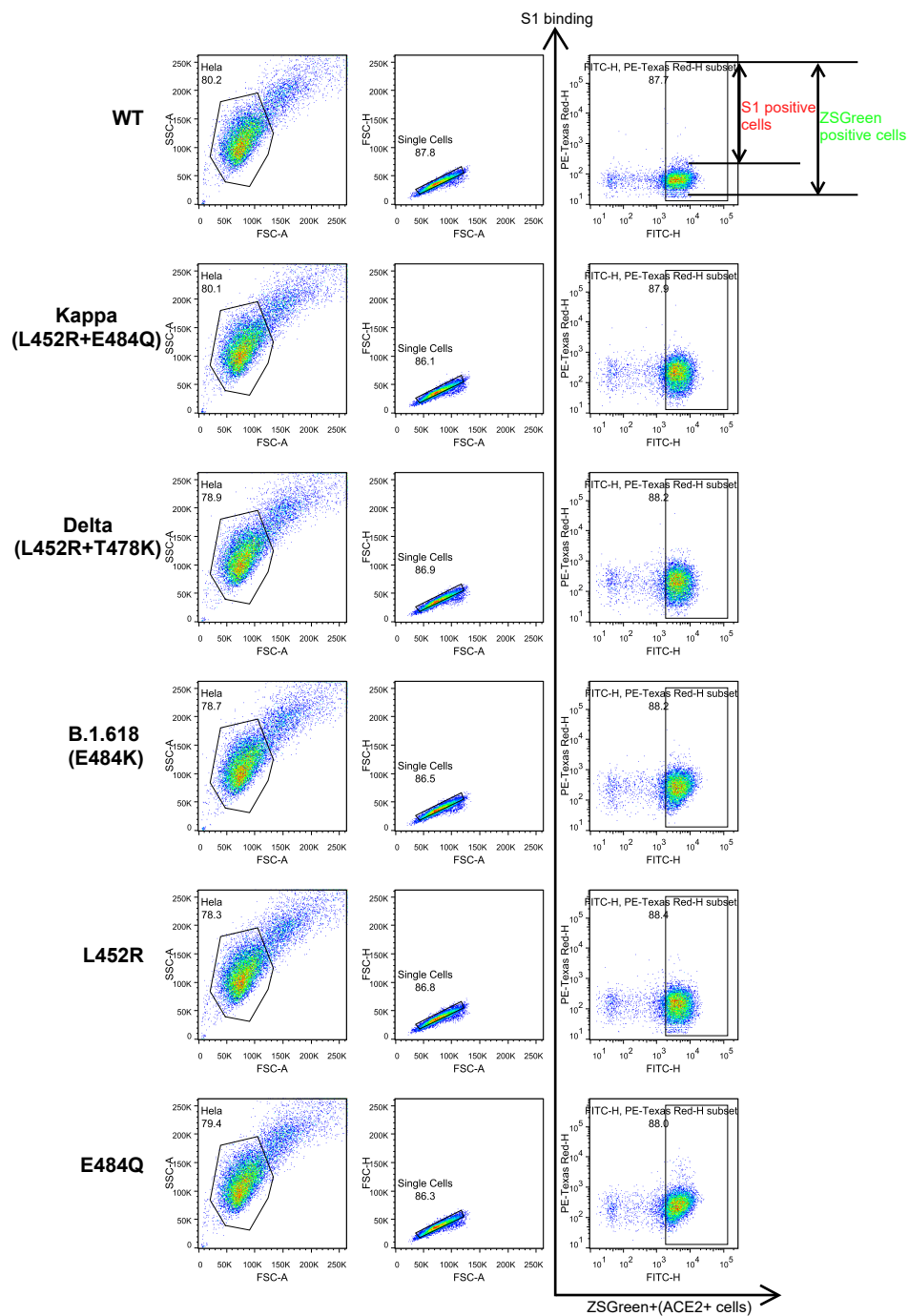

Supplement: FIG S1 [file mbio.00099-22-sf001.pdf]

Supplemental Fig 2

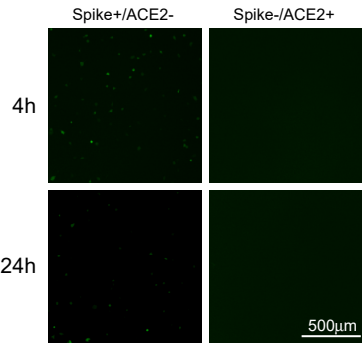

Supplement: FIG S2 [file mbio.00099-22-sf002.pdf]
